# Supplementary material for: Spin-polarized edge modes between different magnet-superconductor-hybrids
Source: Nat Commun. 2026 Apr 11;17:3457. doi: 10.1038/s41467-026-71687-3 (PMC13076643; doi:10.1038/s41467-026-71687-3)
Supplement: Supplementary file 1 — Supplementary Information [file 41467_2026_71687_MOESM1_ESM.pdf]

Supplementary Information for:
Spin-polarized edge modes between different magnet-superconductor-hybrids

Felix Zahner ◇,[1,*] Felix Nickel ◇,[2,†] Roberto Lo Conte,[3] Tim Drevelow,[2]
Roland Wiesendanger,[1] Stefan Heinze,[2,4] and Kirsten von Bergmann[1]

[1]*Department of Physics, University of Hamburg, Jungiusstraße 11, 20355 Hamburg, Germany*
[2]*Institut für Theoretische Physik und Astrophysik, Christian-Albrechts-Universität zu Kiel, D-24098 Kiel, Germany*
[3]*Zernike Institute for Advanced Materials, University of Groningen, The Netherlands*
[4]*Kiel Nano, Surface, and Interface Science (KiNSIS), University of Kiel, Germany*
◇ *These authors contributed equally: Felix Zahner, Felix Nickel.*

EXPERIMENTS

Supplementary Note 1: Magnetization Direction

To identify the magnetization axis of the antiferromagnetic (AFM) states in the Mn monolayer (ML) and Mn bilayer (BL) we use a soft-magnetic Fe/W-tip that typically has an in-plane magnetization in zero field, and aligns its magnetization with applied magnetic field. At zero field we observe no magnetic contrast, see Fig. S1a,b for a constant-current topographic image and current map, respectively. Note that the periodic oscillation in the Mn ML near the buried Ta step edge (upper center of Fig. S1c) originate from standing electron waves with a period of about 0.9 nm at this bias voltage. Upon applying an out-of-plane magnetic field of $+2.5$ T the fine lines, indicative of the AFM state, are seen in both the Mn ML and Mn DL, see Fig. S1b. At this value of applied magnetic field the tip magnetization aligns itself with the magnetic field and is therefore out of plane. Reversing the field to -2.5 T shows an inversion of the magnetic line contrast, see Fig. S1d. This inversion of magnetic contrast can be best seen by comparing the interference pattern of the standing electron waves on the ML and the stripe magnetic contrast in b and d.

Supplementary Note 2: Magnetic ground state of the Mn ML.

The ground state of the extended Mn ML is a spin spiral, where the magnetic moments rotate along $[001]$, similar to the one in the Cr monolayer on $\mathrm{W}(110)$ [1]. In the $[1\bar{1}0]$-direction the magnetic moments invert from one atomic row to the next, resulting in a line contrast in the SP-STM images. For the undisturbed Mn ML region at the top of Fig. S2, this contrast vanishes periodically as the magnetic moments rotate to be oriented perpendicular to the tip magnetization. After about 18 nm one spin spiral period is completed. However, in small Mn ML patches and in particular in the vicinity to the Mn BL the rotation of the spin spiral is suppressed and the magnetic state is a collinear AFM state, see lower region in Fig. S2. The two layers have a strict phase relation between them, suggesting strong antiferromagnetic interlayer exchange coupling between the two AFM states, see inset in Fig. S2b.

Supplementary Note 3: Mn ML bulk in-gap states

To investigate the effect of the rotation of the AFM spin spiral on the superconducting gap of the Mn ML we measured equally spaced tunneling point spectra along the 30 nm long red line marked in Fig. S3a. The result is plotted as a waterfall-plot in Fig. S3b. We do not observe any modulation inside the gap across distances larger than the spin-spiral period (18.5 nm), indicating that in-gap states for the in-plane and out-of-plane areas of the AFM spin spiral do not differ measurably. This is different for the Fe/Ta(110) system were a ferromagnetic spin spiral was found to lead to a modulation inside the superconducting gap with the spin-spiral period [2].

Supplementary Note 4: Mn ML edge state

To investigate the edge mode at the edges between Mn ML and Ta substrate, we measure the $\mathrm{d}I/\mathrm{d}U$ signal at zero bias by first tracing the topography at a bias voltage outside the superconducting gap, typically 4 mV, and then replaying this topography signal for the zero bias measurement (also called multi-pass). We can show that the measured edge mode of the Mn ML is related to the superconductivity, as the edge mode contrast vanishes when the superconductivity is quenched at 200 mT, but reappears upon switching off the magnetic field, see Fig. S4. The measured intensity at zero bias at the edge depends on the edge direction. We observe the highest intensity at the $[001]$ edges and a much lower intensity at the $[1\bar{1}1]$ and $[1\bar{1}0]$ edges. Comparing point spectra taken on the different edges as well as the bare Ta and the Mn ML we find a clear peak for the $[001]$ edge close to zero bias for both the sample area shown in the main text Fig. 2 as well as for the sample area shown in Fig. S4a.

Supplementary Note 5: Mn BL morphology

The thin film morphology of the 1.25 AL sample (see Fig. 3 main text) is shown in Fig. S5. Here the SP-STM current map from Fig. 3 in the main text is shown next to the corresponding constant-current topography image. Estimating the position

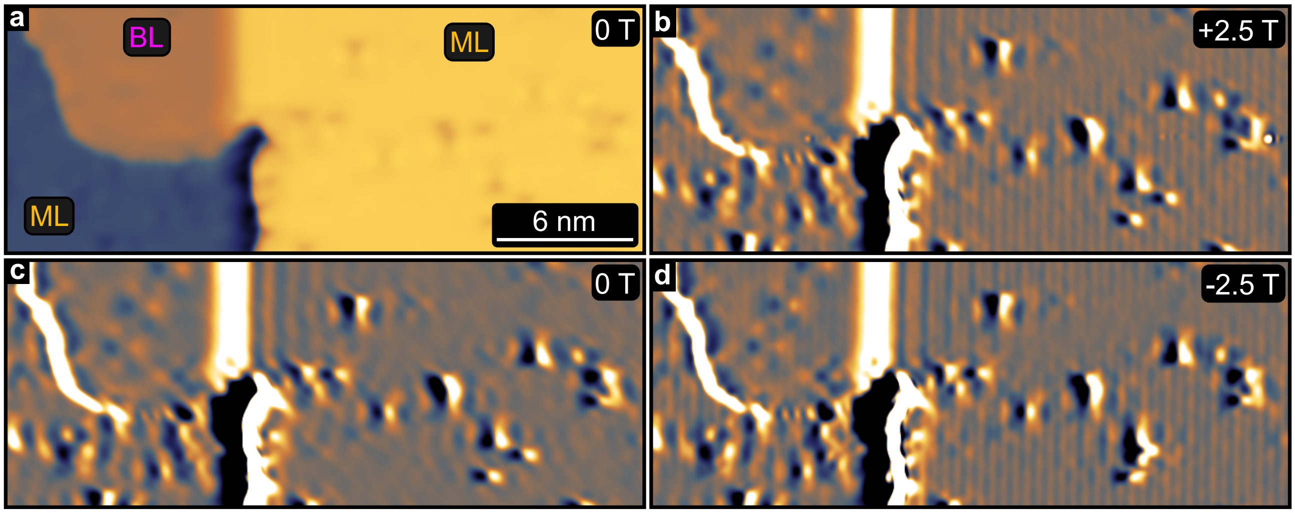

FIG. S1. **Magnetization Direction..** **a,** STM topography image of a Mn BL area (top left) next to the Mn ML. **b-d,** STM current maps of the same area at the magnetic filed values indicated; because a soft magnetic Fe/W-tip is used the tip magnetization direction changes from in-plane in c, to opposite out-of-plane in b,d. High frequency noise was removed by low-pass filtering. Measurement parameters for all: $U = 10\,\mathrm{mV}$, $I = 1\,\mathrm{nA}$, Fe-coated W tip, $T = 8.7\,\mathrm{K}$.

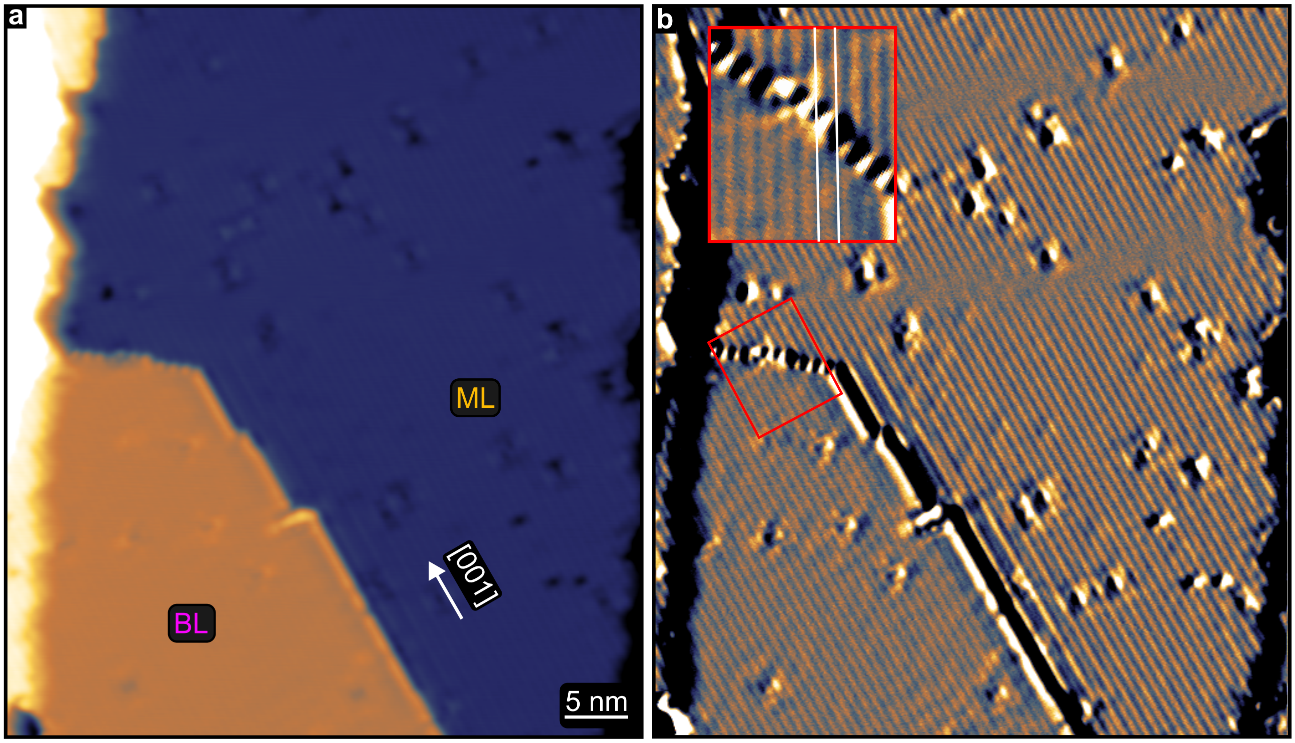

FIG. S2. **Magnetic ground state of the Mn ML.** **a,** STM constant-current topography image of a Mn BL area (bottom left) next to the Mn ML(right). **b,** Simultaneously measured STM current map of the same area showing magnetic contrast on both the BL and ML regions. Inset is a zoom-in of the area in the red rectangle. Measurement parameters for both: $U = -15\,\mathrm{mV}$, $I = 2\,\mathrm{nA}$, Cr bulk tip, $T = 4.2\,\mathrm{K}$.

of the last row of atoms at the edge was done by first comparing the experimental topography lineprofile to a simulated constant current lineprofile, see Fig. S6. Using the magnetic contrast on the BL or ML one can align the atom rows with the lineprofile, see Fig. S6b. As the simulation clearly shows a reduction in the topography signal even before the last atomic row, see Fig. S6c, we find the most likely position of the last atomic row in the experimental lineprofile, see solid red vertical line in Fig. S6b. Doing this for the $\mathrm{d}I/\mathrm{d}U$-maps taken across the oppositely magnetized $[001]$ BL edges we can find the best estimate for the position of the last atomic row in the BL also for the $\mathrm{d}I/\mathrm{d}U$ signal, see Fig. S7c. Here one needs

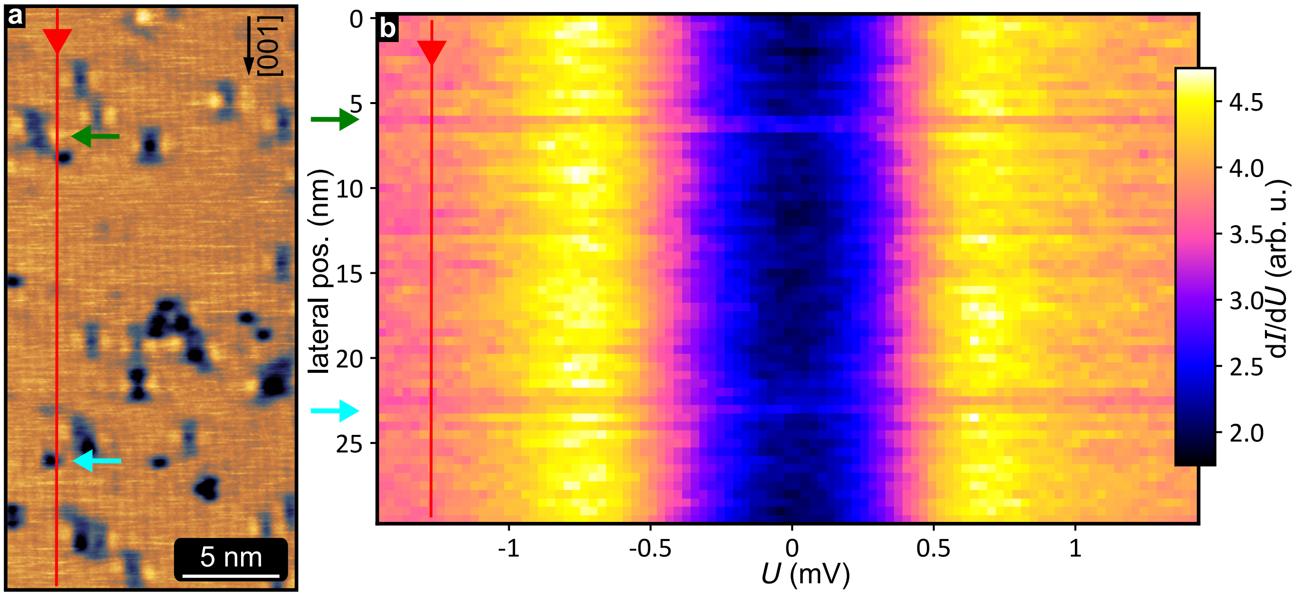

FIG. S3. **Tunneling spectra along a spin-spiral period. a,** STM topography image of a Mn ML region showing the presence of the AFM spin-spiral with a period of 18.5 nm. $U = 4\,\text{mV}$, $I = 1\,\text{nA}$. **b,** 60 tunneling point spectra were taken along a 30 nm long red line shown in **a**. The green and cyan arrows indicate spectra taken near the defects marked by similar arrows in a. All spectra were taken with the following parameters: stabilization bias $U_\text{s} = 4\,\text{mV}$, stabilization current $I_\text{s} = 1\,\text{nA}$, modulation bias $U_\text{mod} = 50\,\mu\text{V}$, Cr bulk tip, $T = 1.3\,\text{K}$.

to adjust for the delay of the $\mathrm{d}I/\mathrm{d}U$ signal due to the lock-in technique. A similar procedure can be done for the tunnel spectra taken across the edge by using the tip stabilization position recorded for each spectrum. The raw data from Fig. 4d,e in the main text is shown in Fig. S7a,b. For the figure in the main text each spectrum was normalized using the y-intercept of a line fitted to the data outside the gap.

Supplementary Note 6: Mn BL edge state

Similar to the edge mode at the boundary between Mn ML and Ta we find that the edge mode between Mn BL and Mn ML vanishes at an applied magnetic field of 200 mT, see Fig. S8. For each field value in Fig. S8b-d we show the topography, the zero bias multi-pass map and the zero bias constant height map. One can see an inversion in contrast in the topography for 200 mT compared to 0 mT seemingly indicating that the z-component of the tip magnetization has changed. This does, however, seem to happen at an unexpectedly low field. It seems more likely that the spin-polarization of the states near the Fermi energy is different for the superconducting and non-superconducting case. While we observe no contrast at the edge for the multi-pass mode at 200 mT we do observe a non-vanishing spin-polarized contrast in the constant height data at zero bias even when the superconductivity is quenched. This contrast is just a measurement of the spin-polarized LDOS near the Fermi-energy and similar to the contrast measured at the edges at a bias of 5 mV, see Fig. S5a. The difference between multi-pass and constant height data is not unexpected as the changing tip-sample distance in the multi-pass modes may exactly compensate this spin-polarization resulting in no observable contrast.

We performed zero bias measurements at the boundary between the Mn BL and the bare Ta substrate, see Fig S9. Constant height measurements across the vertical straight [001] edges show a strong $\mathrm{d}I/\mathrm{d}U$ signal at zero bias localized at the edge, see Fig. S9b,c. As we know the Ta substrate is in a trivial state, we conclude that the Mn BL is in a TNPSC phase. We have not observed straight edges between the Mn BL and Ta substrate along any other direction in our samples.

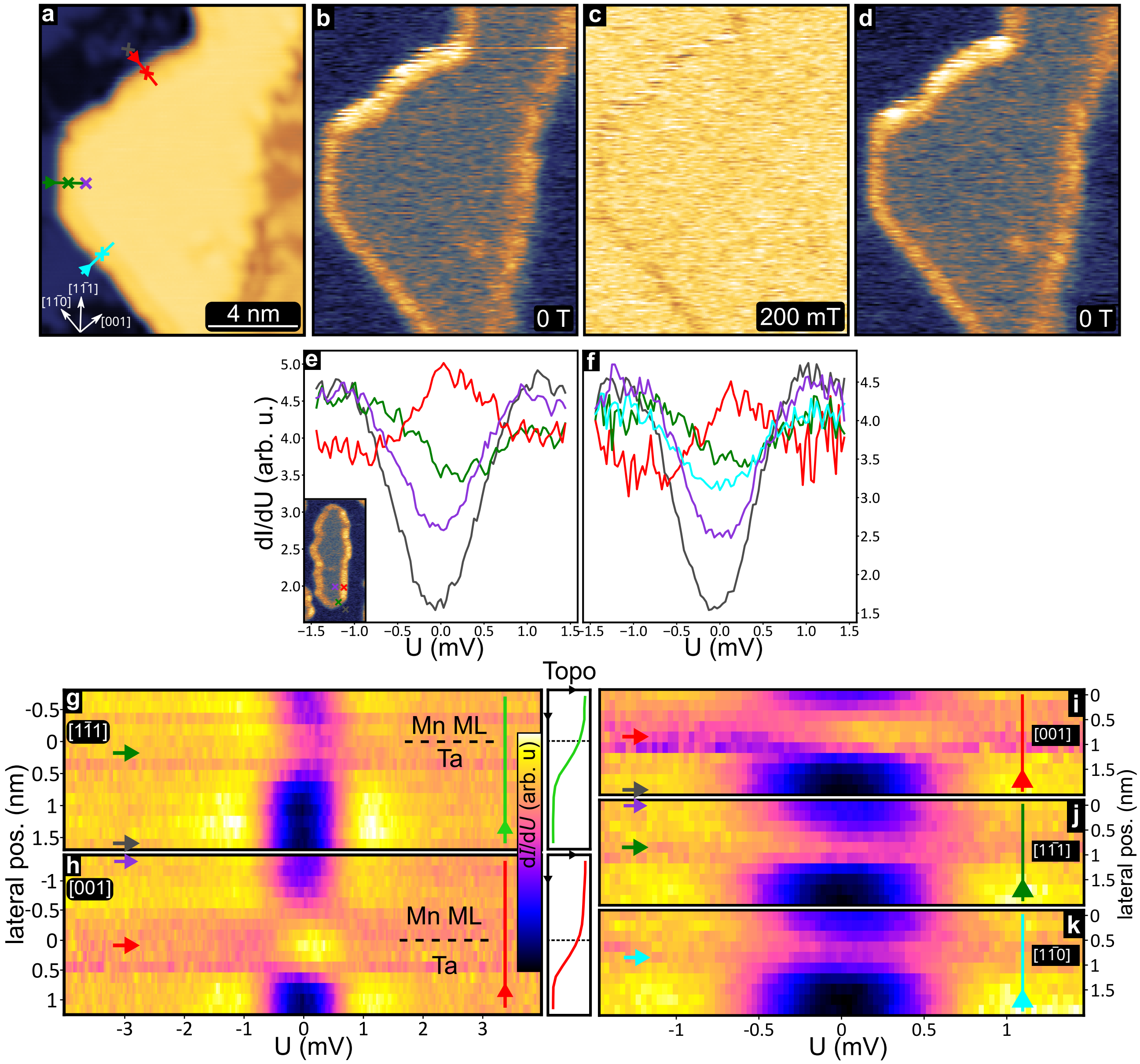

FIG. S4. **Mn ML edge state. a,** STM topography image of a Mn ML island grown from a Ta step edge. **b-d,** Maps of differential tunnel conductance ($\mathrm{d}I/\mathrm{d}U$) at zero bias of the same area as in (a) measured at the indicated applied magnetic field. **e,** Tunneling point spectra taken at the locations marked in inset (bottom left). Bare Ta (blue), Mn ML island (purple), [001]-edge (red) and $[1\bar{1}\bar{1}]$-edge (green). **f,** Tunneling point spectra taken at the locations marked (cross) in (a). Bare Ta (blue), Mn ML island (purple), [001]-edge (red), $[1\bar{1}1]$-edge (green) and $[1\bar{1}0]$-edge (cyan). **g-h,** Raw data corresponding to the waterfall plots in Fig. 2d,e of the main text. For the main text figure each spectrum was normalized using the y-intercept of a line fitted to the data outside the gap. Graphs on the right show the height the tip was stabilized at before each spectrum, which was used to estimate the position of the last row of atoms at the ML edge; color range $1.5 - 5.0$ arb. units. **i-k,** Waterfall plots of tunnel spectra taken along the colored lines marked in (a). Colored arrows indicate spectra shown in (e) and (f) recorded at the marked locations in (a). Measurement parameters: a-d: $U = 4\,\mathrm{mV}$, $I = 1\,\mathrm{nA}$, $U_{\mathrm{mod}} = 50\,\mu\mathrm{V}$; for all spectra: stabilization bias $U_{\mathrm{s}} = 4\,\mathrm{mV}$, stabilization current $I_{\mathrm{s}} = 1\,\mathrm{nA}$, modulation bias $U_{\mathrm{mod}} = 50\,\mu\mathrm{V}$; all: Cr bulk tip, $T = 1.3\,\mathrm{K}$.

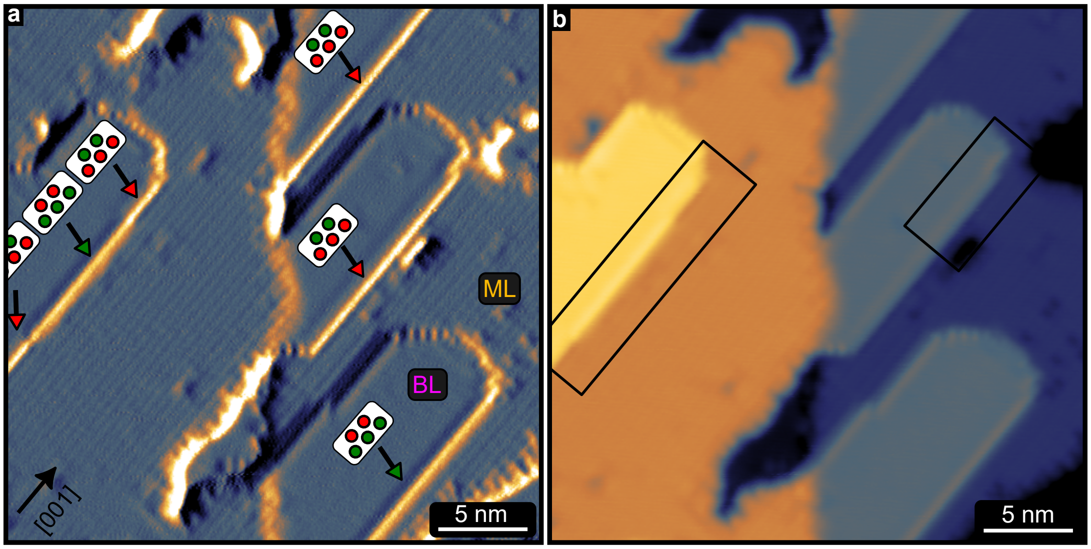

FIG. S5. **BL thin film morphology.** **a,** SP-STM current map of 1.25 AL of Mn on Ta(110), directly corresponding to Fig. 3a in the main text. **b,** Constant-current STM image of the same region as in a. Black rectangles indicate regions shown Fig. 3c,d in the main text. Measurement parameters: a,b: $U = +5\,\text{mV}$, $I = 1\,\text{nA}$, $T = 1.3\,\text{K}$, Cr bulk tip.

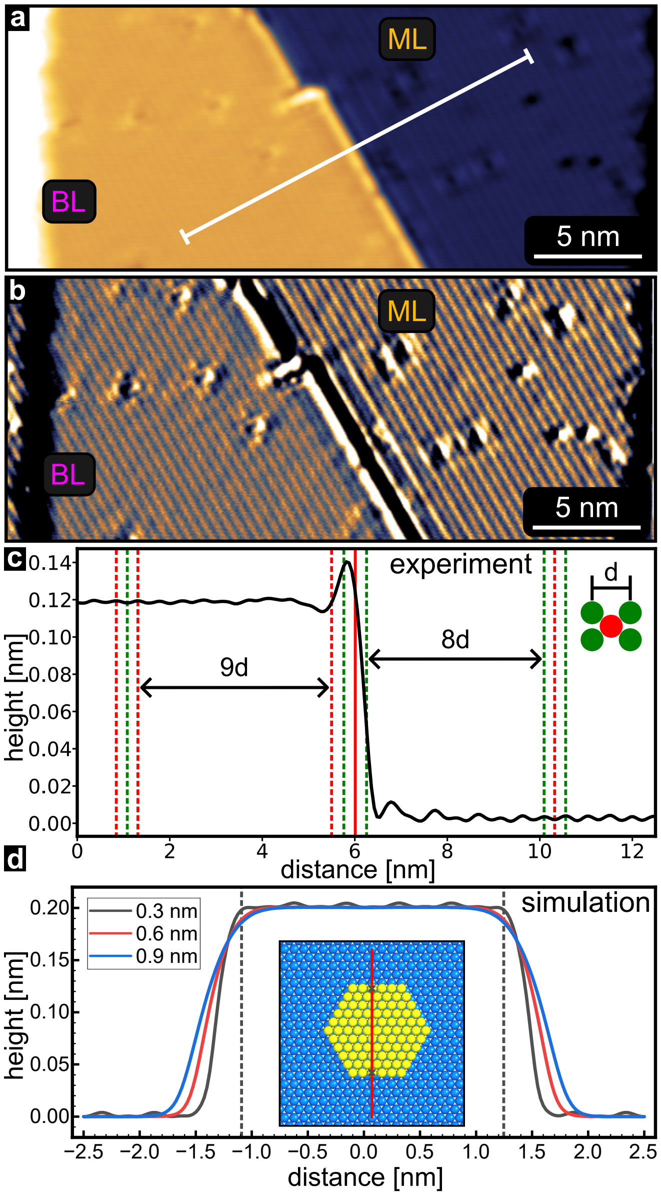

FIG. S6. **BL edge state.** **a-b,** SP-STM topography image and current map of a sample region with BL on the left and ML on the right. $U = -15\,\mathrm{mV}$, $I = 5\,\mathrm{nA}$, $T = 4.2\,\mathrm{K}$. **c,** Lineprofile taken along the white line in **a**. The dashed red/green vertical lines mark the position of some of the atomic rows. The solid red line shows the best estimate of the position of the center of the last atomic row of Mn BL atoms. **d,** Height profiles from constant-current STM simulations across a hexagonal island (see red path in inset) for different tip-sample distances. The vertical grey lines represent the position of the center of the last row of BL atoms. The simulations were done by André Kubetzka using a single atom s-wave tip.

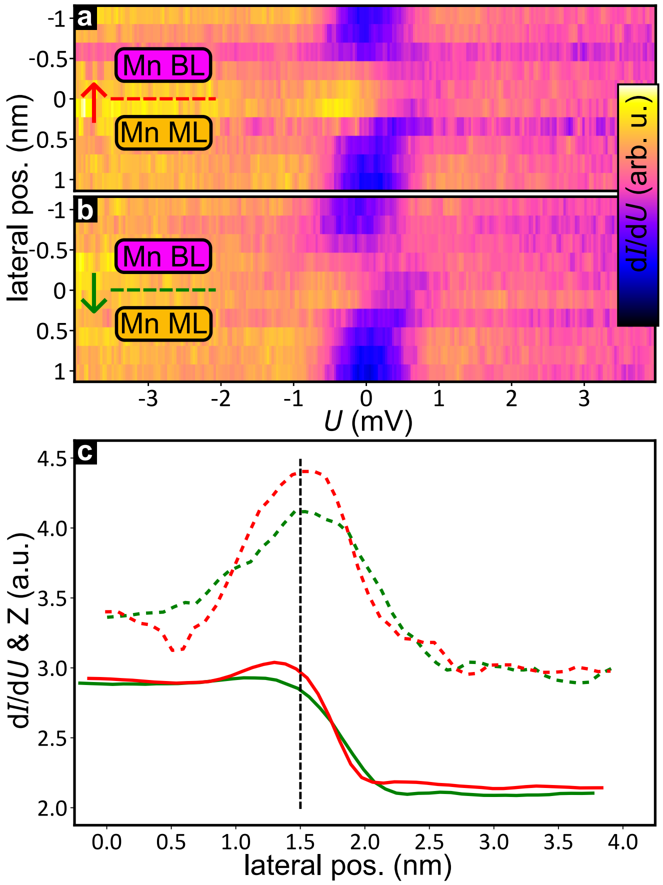

FIG. S7. **BL edge state.** **a-b,** Waterfall plots of tunneling spectra taken across two BL-ML edges with opposite magnetization., directly corresponding to Fig. 4c,d in the main text and plotted using the raw data here; color range $2.5 - 5.0$ arb. units. Experimental parameters: stabilization bias $U_\mathrm{s} = 4\,\mathrm{mV}$, stabilization current $I_\mathrm{s} = 1\,\mathrm{nA}$, modulation bias $U_\mathrm{mod} = 50\,\mu\mathrm{V}$. **c,** Line profiles of the same edges measured in a $\mathrm{d}I/\mathrm{d}U$ map by retracing the topography measured at 4 mV. After adjusting for lock-in delay we can estimate the position of the last row of Mn atoms in the BL using the topography line profiles and compare them to the $\mathrm{d}I/\mathrm{d}U$ line profiles plotted using full/dotted lines respectively.

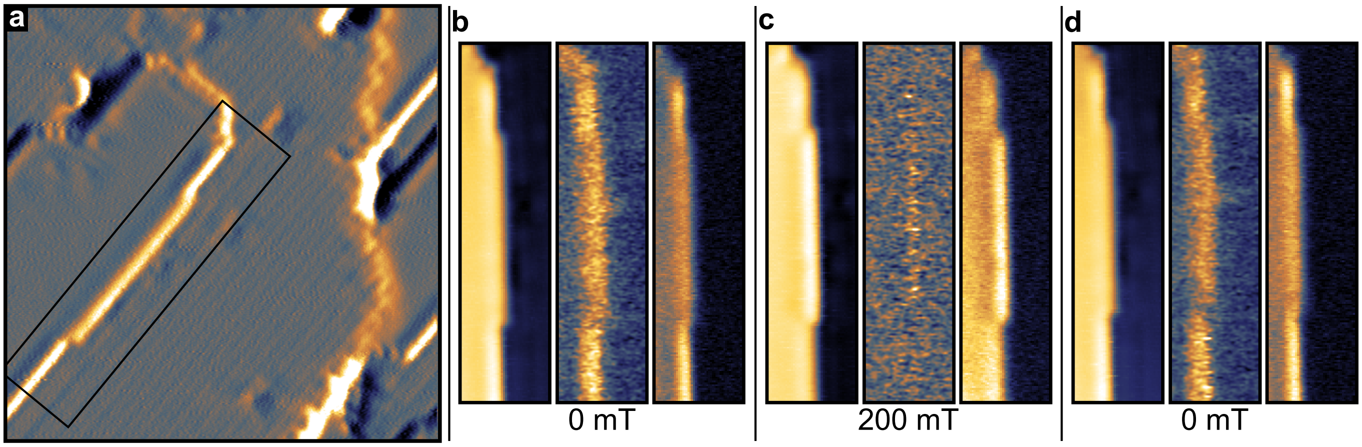

FIG. S8. **BL edge state in magnetic field. a,** SP-STM current image of an area of Mn BL (left) next to Mn ML; $U = 5\,\text{mV}$, $I = 1\,\text{nA}$. **b-d,** Mn BL edge along the [001] direction at 0 mT and 200 mT. For each field value the topography at 4 mV and 1 nA (left), $\mathrm{d}I/\mathrm{d}U$ at 0 mV (multi-pass, middle) and $\mathrm{d}I/\mathrm{d}U$ at 0 mV (constant height, right) are shown. All the $\mathrm{d}I/\mathrm{d}U$ maps were recorded with a bias modulation of 50 μV.

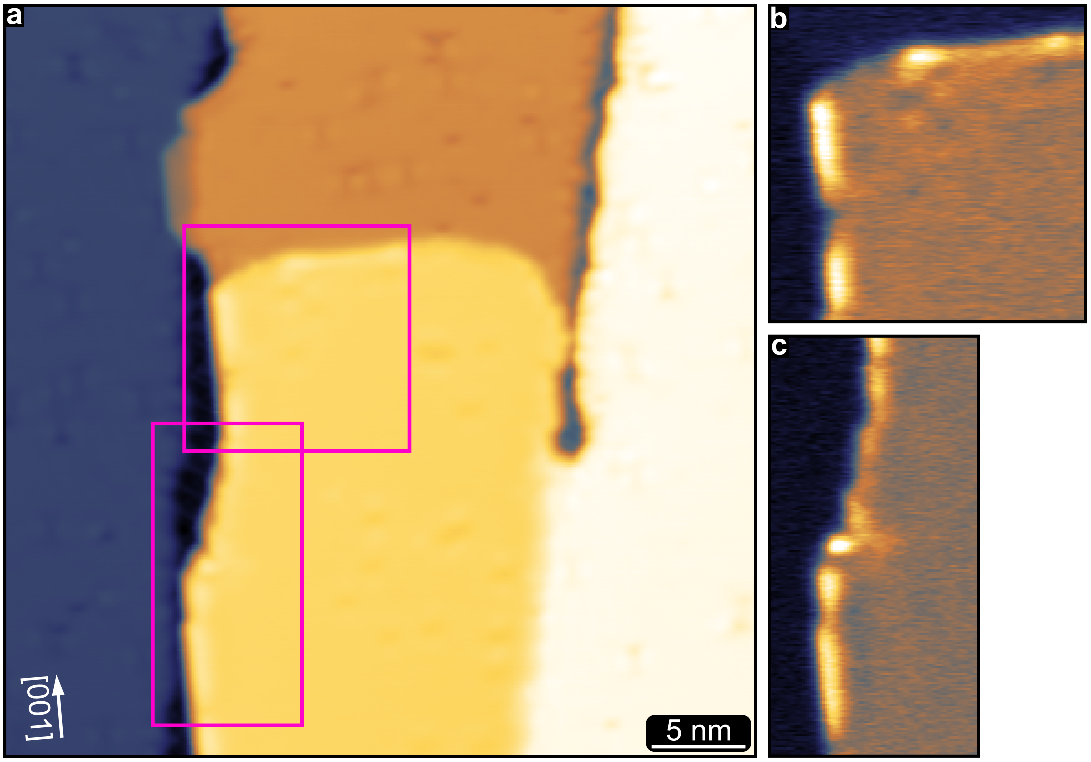

FIG. S9. **BL edge state at BL-Ta edge. a,** STM topography image of 1.25 ML of Mn on Ta(110). The regions within the pink rectangles show vertical edges from the BL directly to the bare Ta substrate running along the [001] direction. $U = 15\,\text{mV}$, $I = 300\,\text{pA}$. **b-c,** Constant height $\mathrm{d}I/\mathrm{d}U$ images measured in the regions of the pink rectangles in **a** at 0 mV. The tip was stabilized at 4 mV, 1 nA.

ML		BL	
		$Mn_{top} - Mn_{bottom}$	1.430
$Mn - Ta_1$	1.869	$Mn_{bottom} - Ta_1$	1.857
$Ta_1 - Ta_2$	2.247	$Ta_1 - Ta_2$	2.237

Supplementary Table 1. **Interlayer distances for the Mn ML and BL on Ta(110).** The interlayer distances obtained from structural relaxations performed in DFT calculations between the Mn layers and the two topmost Ta layers of the surface. All values are given in Å.

ML		BL	
		Mn_{top}	2.74
Mn	2.78	Mn_{bottom}	1.03
Ta_1	0.21	Ta_1	0.15
Ta_2	0.02	Ta_2	0.04

Supplementary Table 2. **Magnetic moments for the Mn ML and BL on Ta(110).** The magnetic moments calculated via DFT are given for the Mn atoms (top and bottom layer for the BL) and for the two upmost Ta layers of the surface. All values are given in μ_B.

THEORY

Supplementary Note 7: DFT calculations

Based on density functional theory (DFT) we have calculated the Ta bulk lattice constant and performed structural relaxations of the Mn ML and Mn BL on the Ta(110) surface using the VASP code [3–6] and applying the PBE [7] GGA exchange-correlation functional. The obtained theoretical Ta lattice constant was 3.321 Å which deviates by only 0.6% from the experimental value of 3.30 Å. Based on this lattice constant, a symmetric slab made from 13 Ta layers and with a (110) surface and a Mn ML/BL on each side was structurally relaxed to determine the interlayer distances. Relaxations were performed for the FM state and the RW-AFM state in each layer. For the BL, there was always an antiferromagnetic alignment between two Mn layers. The RW-AFM configurations are always preferred, by 243 meV/unit cell for the ML and 173 meV/unit cell for the BL. The interlayer distances between the Mn layers and the topmost Ta layers of the surface are given in Supplementary Table 1 for the RW-AFM states of ML and BL. The magnetic moments obtained for the Mn and Ta surface layers are given in Supplementary Table 2. The calculated work function is 4.23 eV for the Mn ML on Ta(110) and 4.45 eV for the Mn BL on Ta(110).

Supplementary Note 8: Tip spin polarization

For the SP-STM simulations shown in the main text (Fig. 8) we assumed a 100% spin polarization of the tip. Fig. S12 shows the same type of simulations as in the main text, but with a 50% spin polarization. In Fig. S12d the ↑ and the ↓ contributions approach the spin-averaged value. This leads to a small peak also in the ↓ signal close to the maximum of the spin polarization. The spin polarization in Fig. S12e displays a maximum value of 27% which is around half the value obtained for the 100% spin polarized tip cf. Fig. 8 of the main text). If one would assume a 7% tip polarization, the peak of the spin polarization would have about the same height as in the experiment. Note, that in this comparison the spin polarization incorporates the effect of a non-collinear alignment of the tip and sample magnetization direction. Therefore, the small value of the effective spin polarization needed for a quantitative agreement with the experiment still seems reasonable.

Supplementary Note 9: Lateral decay of the vacuum LDOS

In the main text the decay parameter is calculated for each surface state by the complex band structure (cf. Fig. 9). Further exponential functions are fitted to the spin-averaged LDOS at the lattice site. Here we discuss the fit to the lateral decay of the vacuum LDOS of the spin-up, spin-down and spin averaged data. Fig. S15 shows the integrated vacuum LDOS from Fig. S14. For each direction from the boundary we fit the function

$$A = A_0 \, \mathrm{e}^{-\kappa^{\mathrm{eff}}(x-x_0)} + C \tag{1}$$

to the vacuum LDOS. Here x represents the distance, x_0 is the position of the maximal value, A_0 is the amplitude and C an offset. The lateral decay of the vacuum LDOS towards the side of the BL or the ML is very similar to that at the lattice sites shown in the main text (Fig. 9c). The lateral decay parameter into the ML is around 0.36 and larger than that for the BL which is 0.26. Furthermore, the values are very similar between both spin channels and the spin-averaged signal. As expected from the complex band structure, the lateral decay seems not to depend on the spin channel. Also the decay of the edge state into the vacuum does not affect the lateral decay into the BL or ML. This shows that the intrinsic decay from the edge state is also detectable by STM.

Supplementary Note 10: transformation of nodal points

Topological nodal points are protected against spontaneous annihilation. The ML and BL are simulated by sets of parameters, which differ only in the value of JS. Therefore both systems can be transformed into each other by a continuous transformation of JS from $JS_{\mathrm{BL}} = 2.5$ to $JS_{\mathrm{BL}} = 4.0$. In Fig. S17 the positions of bulk nodal points are marked in the 2D-BZ, where the color encodes the value of JS. When the parameter is varied from 2.5 to 4.0 the nodal points on the boundary of the BZ shift further to the corner. These nodal points are considered equivalent nodal points, as they can be transformed into each other. At a critical value of $JS = 2.9$ two new nodal points form in the center of the BZ and split

for an increased value of JS. These new nodal points, which exist in the ML have no equivalent correspondent in the BL.

The edge modes, which were observed in Fig. 7 of the main text, form between the equivalent nodal points on the boundary of the BZ. This shows, that edge modes cannot only occur, if the number of nodal points changes, but also if the nodal points are just slightly shifted in one domain. To test the robustness of the edge mode observed in Fig. 7 of the main text, Fig. S21 shows the band structure for a stripe geometry in the $[001]$ direction, where one domain is formed by the BL and in the other domain the parameter JS is varied. Fig. S21p corresponds to the same band structure as in the main text and Fig. S21a resembles the BL bulk band structure. For a value of $JS_{\mathrm{mod}} = 2.6$ the nodal points become wider and even for $JS_{\mathrm{mod}} = 2.7$ four distinct nodal points with edge states between them can be observed. For further increasing values of JS, the nodal points are separating more, while the edge mode is still connecting two equivalent nodal points. The emerging nodal points in the middle of the BZ for $JS \geq 2.9$ are not involved in the edge mode formation. Beside the edge modes in the $[001]$ direction, also a stripe geometry in $[1\bar{1}0]$ direction shows edge modes.

The band structure for a stripe geometry of alternating ML and BL stripes, equivalent to Fig. 7 of the main text, is shown in Fig. S11a. An edge mode connects the two bulk nodal points of the ML, indicated by yellow vertical lines. Differing for the stripes in $[001]$ direction, here two nodal points are connected which belong to the same domain. The spectral function at the boundary for electronic spin-up ($\uparrow$) and spin-down ($\downarrow$) orbitals are shown in Fig. S11b,c. Here it can be seen that the mode is indeed an edge mode and that it is spin-polarized. Thereby, it can be concluded that a spin-polarization of the edge mode can also occur when two nodal points of the same domain are connected as at the boundary to a trivial state. Fig. S11d summarizes the emerging edge modes from Fig. 7 of the main text and Fig. S11a.

The $[1\bar{1}0]$ boundary between the ML and the BL has an edge mode, similar to the edge mode between the ML and the bare Ta surface (cf. Fig. 6 of the main text). The ML nodal points in the middle of the BZ have no equivalent partner in the BL, as both nodal points meet and retract from the Fermi surface at $JS = 2.9$. The edge modes between two TNPSCs can be of two types: (i) edge modes between equivalent nodal points (nodal points that can be transformed into each other) and (ii) edge modes between a pair of nodal points of one domain, when no equivalent nodal points exist. These edge modes also occur for boundaries to trivial states, as they host no topological nodal points and subsequently can not have equivalent nodal points. The novel implications of the first mechanism are that only a small local disturbance of one MSH system can lead to the formation of edge modes between perturbed and unperturbed regions. A spin polarization of the edge mode is a general effect that can be found in both scenarios. Both mechanisms can also be transformed into each other as shown in Fig. S18. Here, a stripe geometry is shown in $[1\bar{1}0]$ direction for a ML and a modified layer similar to Fig. S21. The parameter JS in the modified domain is varied in the range $2.5 \leq JS_{\mathrm{mod}} \leq 4.0$. Fig. S21a corresponds to the ML bulk. For increasing JS_{mod} the nodal points split and are connected by an edge mode of mechanism (i). At the value of $JS_{\mathrm{mod}} = 2.9$, two edge modes meet and vanish as JS_{mod}. Now the edge modes are connected and form a single edge mode connecting a pair of nodal points following mechanism (ii).

Supplementary Note 11: Choice of TB parameters

For the tight-binding (TB) calculations, we have used the hopping parameter t and the Rashba-SOC parameter α according to Ref. [8]. The superconducting order parameter has been set to $\Delta = 1$. For other spin structures it has been shown that the results are qualitatively robust against the superconducting order parameter [9, 10]. For the chemical potential μ and the coupling to the magnetic state JS three considerations were made: (1) The number of nodal points in the ML and the BL should differ. (2) The number of states in the band gap of the ML should change as little as possible for an orientation of the magnetic moments in the AFM state chosen along the $[001]$ (in-plane) and the $[110]$ (out-of-plane) direction. (3) The number of states for $JS \neq 0$ should be small compared to the band gap of Ta with $JS = 0$. Note that a different number of nodal points is not a necessary condition for the existence of edge modes as shown in the main text. However, if the ML and BL have a different number of nodal points, both types of edge states (between two ML nodal points and between ML and BL nodal points) can be observed. The second point is motivated by a spin spiral, observed in the experiment, where the $\frac{\mathrm{d}I}{\mathrm{d}U}$ signal is nearly constant along the spin spiral (see Fig. S3). The third condition relies also on experimental observations. Further the ratio of $\frac{JS_{\mathrm{ML}}}{JS_{\mathrm{BL}}} \approx 1.63$ is chosen according to the ratio of magnetic moments found via DFT.

Fig. S20a shows the number of nodal points in the BZ obtained in the tight-binding calculations as a function of chemical potential μ and coupling strength JS. The squared difference in the LDOS for an orientation of the magnetic moments in the AFM state along the $[001]$ and a $[110]$ direction is given by

$$\zeta = \int_{-1}^{1} [n_{[001]}(E) - n_{[110]}(E)]^2 \mathrm{d}E \tag{2}$$

and is displayed in Fig. S20b. The integral over the LDOS within the band gap is calculated via

$$\eta = \int_{-1}^{1} [n_{[110]}(E)]^2 \quad dE. \tag{3}$$

and shown in Fig. S20c. The values ζ and η are smaller for $\mu < 0$ than for $\mu > 0$. For a value of $\mu = -3.8$ two values, $JS_{\mathrm{BL}} = 2.5$ and $JS_{\mathrm{ML}} = 4.0$, can be found with four and six nodal points. Thereby, all three conditions are fulfilled for this set of parameters.

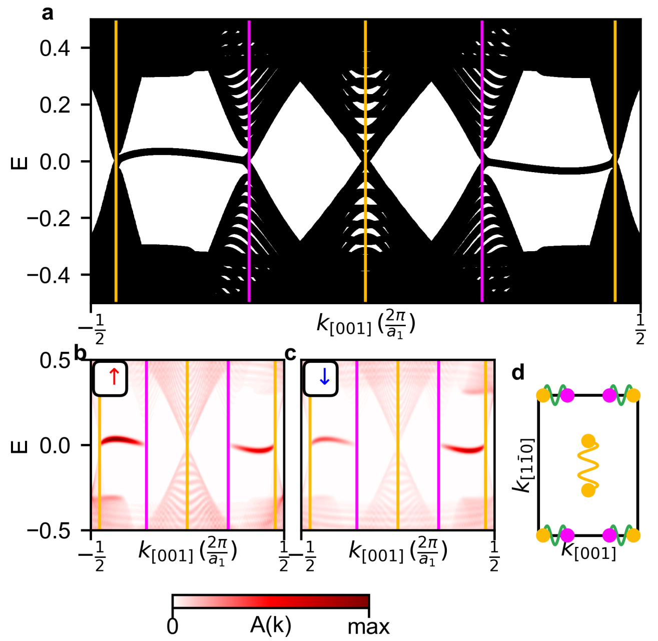

FIG. S10. **Band structure for the ML/BL stripe geometry with an ↑↓ edge.** **a** Band structure along $k_{[001]}$ for ribbons, which are periodic in this direction. The positions of the nodal points from the ML bulk and the BL bulk are marked by vertical lines (cf. Fig- 6 of the main text). **b,c** Spectral functions of the first two rows of the ML and the first two rows of the BL counting from the boundary. The spectral function is shown for the electron spin-up orbitals (**b**) and the electron spin-down orbitals (**c**). **d** Sketches of the 2D-BZ with the positions of the bulk nodal points of the ML and BL. Nodal points which are connected by edge modes for the [001] or $[1\bar{1}0]$ geometry are indicated by wavy lines.

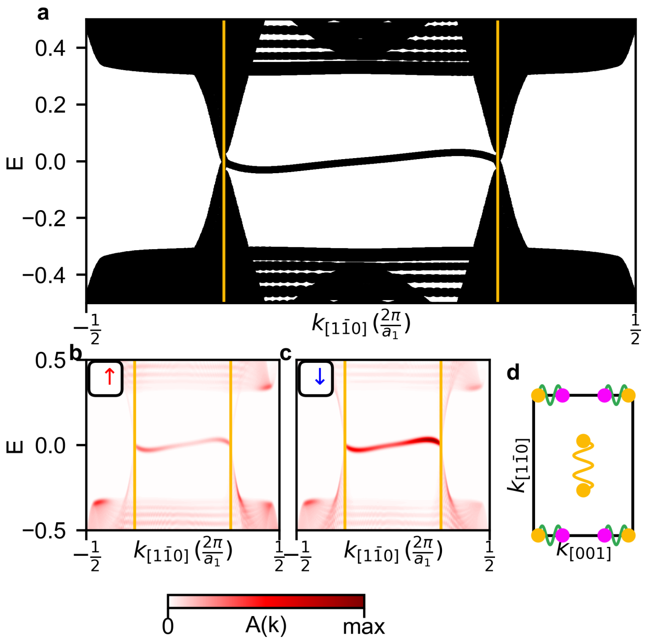

FIG. S11. **Band structure for the ML/BL stripe geometry along** $[1\bar{1}0]$**.** **a** Band structure along $k_{[1\bar{1}0]}$ for ribbons, which are periodic in this direction. The positions of the nodal points from the ML bulk and the BL bulk are marked by vertical lines (cf. Fig- 6 of the main text). **b,c** Spectral functions of the first two rows of the ML and the first two rows of the BL counting from the boundary. The spectral function is shown for the electron spin-up orbitals (**b**) and the electron spin-down orbitals (**c**). **d** Sketches of the 2D-BZ with the positions of the bulk nodal points of the ML and BL. Nodal points, which are connected by edge modes for the [001] or $[1\bar{1}0]$ geometry are connected by wavy lines.

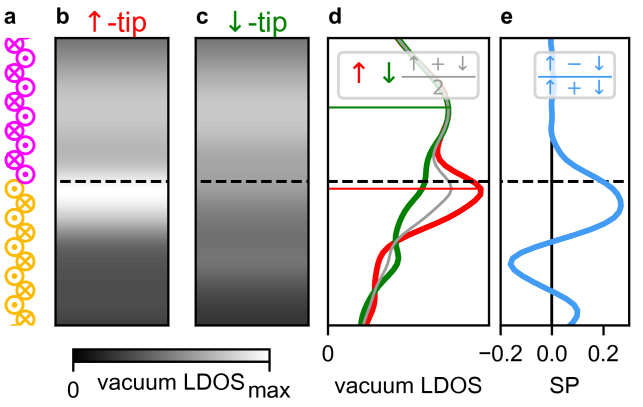

FIG. S12. **Simulated zero-bias SP-STM images for a ↑↑-edge at the ML/BL boundary with** 50% **tip polarization.** **a** Sketch of the magnetic moment for a ↑↑ edge in the [001]-direction between the ML and BL. **b, c** Simulated zero-bias SP-STM contrast for a 100% spin polarized tip, which is ↑ polarized (**b**) or ↓ polarized (**c**). The boundary between both domains is displayed as a black dashed line. The tip height is $4\,\text{Å}$ and the averaged voltage interval is $[-0.02, 0.02]$. **d** Integrated SP-STM signal along [001] for spin-up (red), spin-down (green) and spin-averaged (gray). The position of the maximum intensity is marked by a thin solid line. **e** Spin polarization of the SP-STM signal calculated from the values shown in **d**.

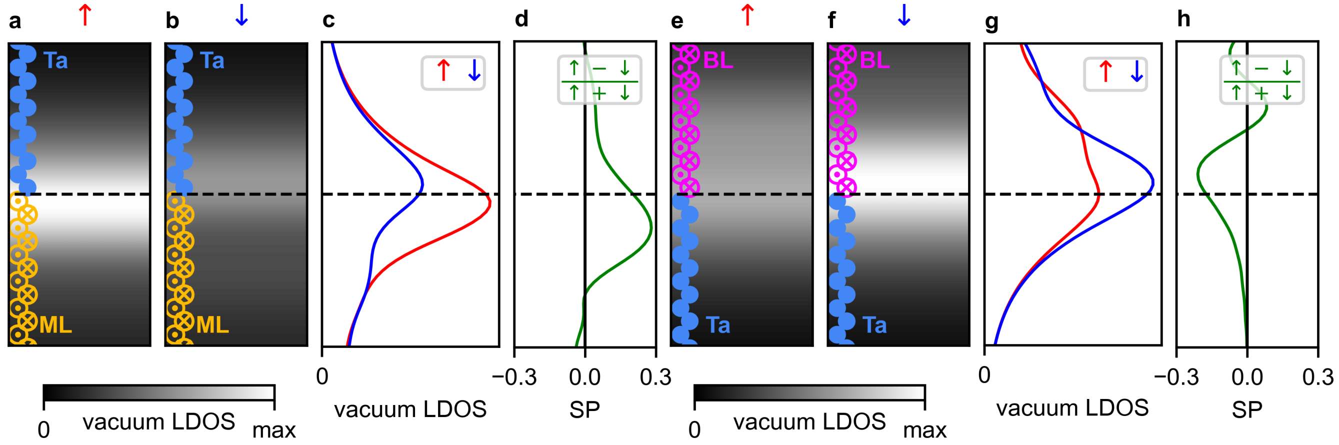

FIG. S13. **SP-STM simulations for the ML/Ta and BL/Ta edge. a-d** Simulations for a boundary between the ML and the bare Ta surface. **e-h** Simulations for a boundary between the BL and the bare Ta surface. **a+e** (**b+f**) show the spin-up (spin-down) contributions. **c+g** display the integrated signal and **d+h** the spin polarization. The simulations were performed for an energy range of $[-0.02, 0.02]$, reflecting zero-bias SP-STM measurements. SP-STM simulations for a ML (a, b, c) and a BL (d, e, f) adjacent to a Ta(110) surface.

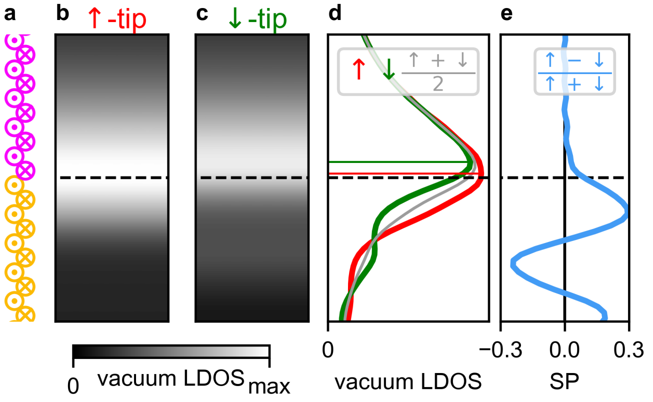

FIG. S14. **Simulated zero-bias SP-STM images for a ↑↓-edge at the ML/BL boundary.** **a** Sketch of the magnetic moments for a ↑↓ edge in the [001]-direction between the ML and BL. **b, c** Simulated zero-bias SP-STM contrast for a 100% spin polarized tip, which is ↑ polarized (**b**) or ↓ polarized (**c**). The boundary between both domains is displayed as a black dashed line. The tip height is 4 Å and the averaged voltage interval is $[-0.02, 0.02]$. **d** Integrated SP-STM signal along [001] for spin-up (red), spin-down (green) and spin-averaged (gray). The position of the maximum intensity is marked by a thin solid line. **e** Spin polarization of the SP-STM signal calculated from the values shown in **d**.

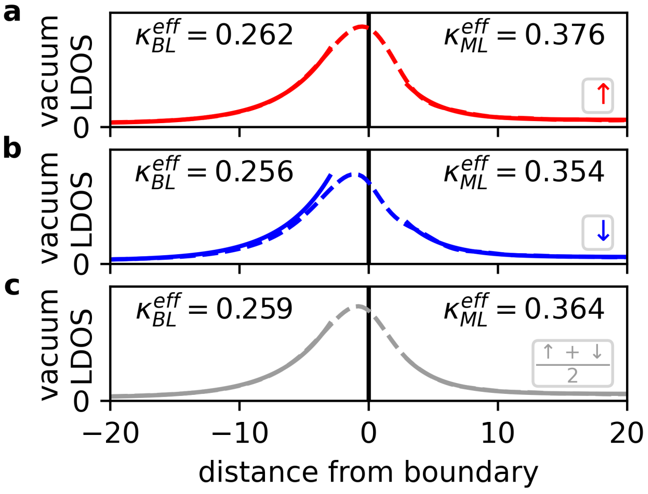

FIG. S15. **Vacuum LDOS for the ↑↓-edge at the ML/BL interface.** The vacuum LDOS from Supplementary Figure S14 is shown for the **a** spin-up channel, **b** spin-down channel and **c** spin averaged by a dashed line. An exponential function is fitted to the signal (solid line) individually in both directions from the boundary. The effective fit parameters are indicated.

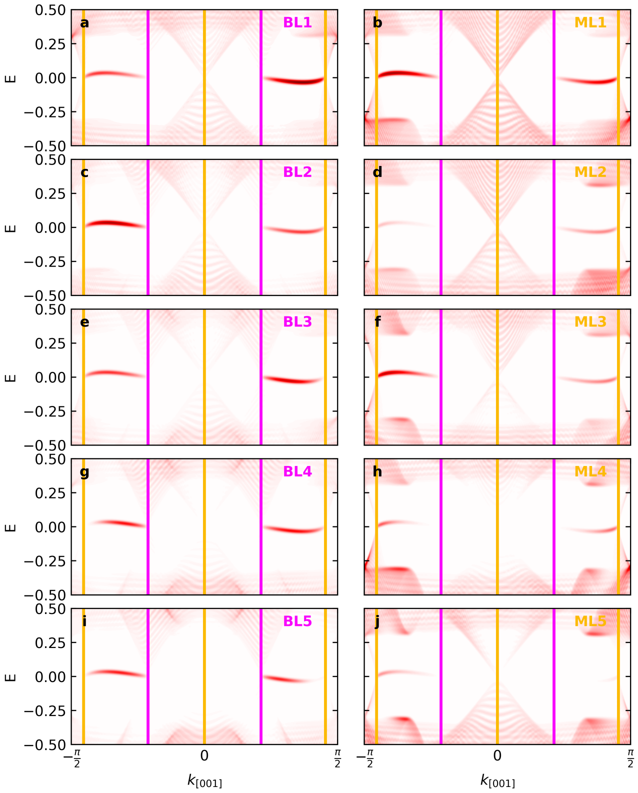

FIG. S16. **Spectral function for the ML/BL boundary with an ↑↓ edge.** The spectral functions of the electronic states (↑ + ↓) are shown for the five BL (**a,c,e,g,i**) and ML (**b,d,f,h,j**) atoms closest to the boundary of a stripe geometry in the $[1\bar{1}0]$ direction. The positions of the bulk nodal point are marked.

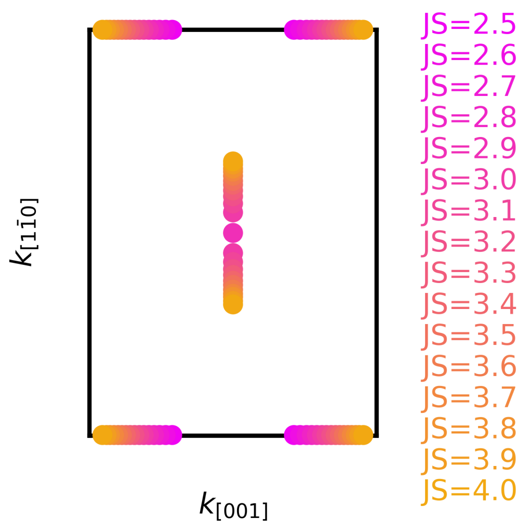

FIG. S17. **Position of nodal points.** The positions of nodal points, obtained from bulk calculations are marked in the 2D-BZ. The coupling to the magnetic layer is varied in the range $2.5 \leq JS \leq 4.0$. All other parameters are as described in the methods section of the main text. The parameters $JS = 2.5$ and $JS = 4.0$ correspond to the BL and ML, respectively. The varying parameters model therefore a continuous transformation from the BL to the ML.

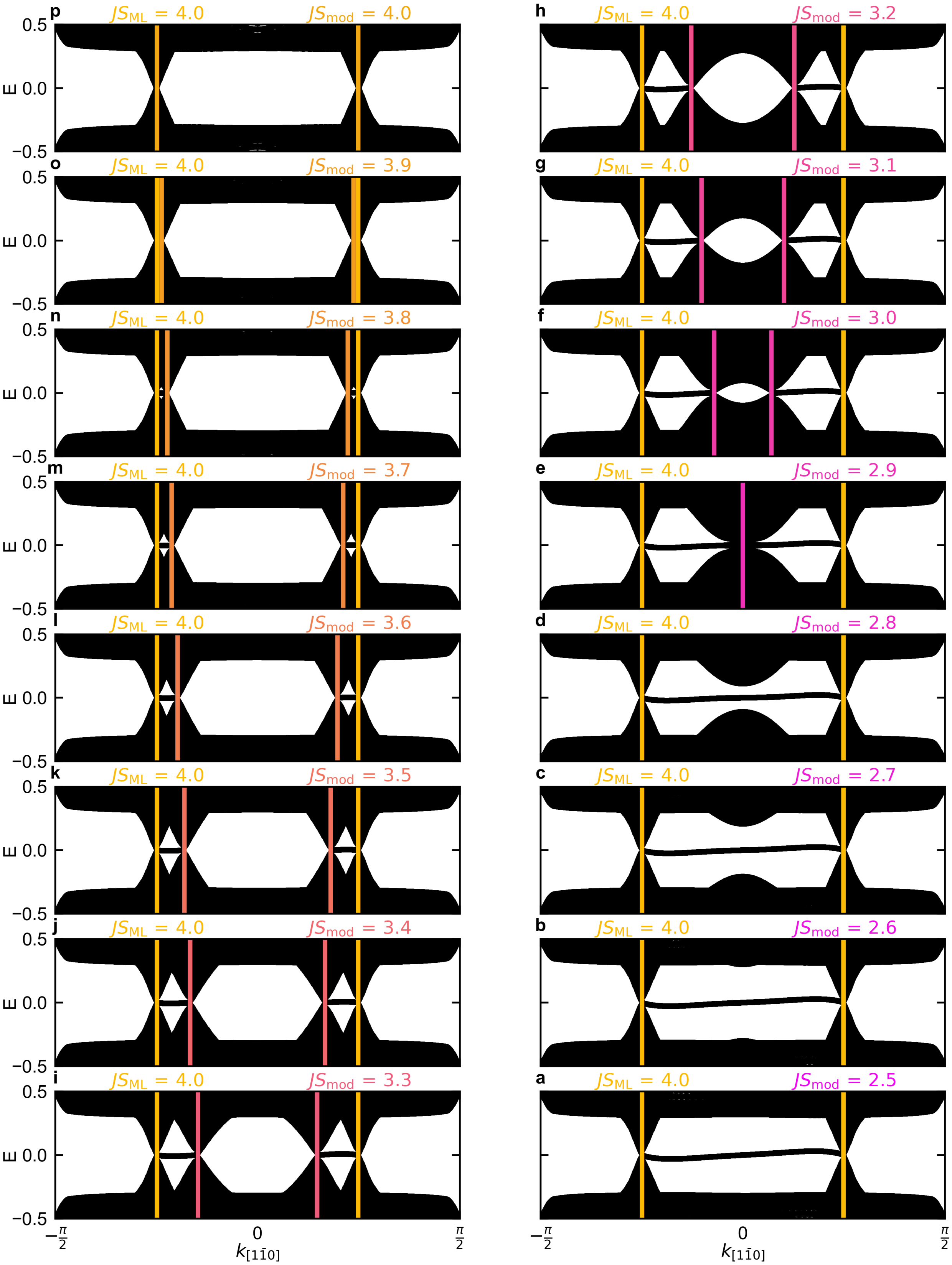

FIG. S18. **Band structures for a stripe geometry with an ↑↓ edge for alternating domains of ML and a modified layer with varied** $2.5 \leq JS_{\mathrm{mod}} \leq 4.0$**.** (**a-p**) The value of JS_{mod} is varied in 0.1 increments. The stripe is oriented along the $[1\bar{1}0]$ direction. The position of the ML bulk nodal points and of the bulk nodal points of the modified layer (cf. Fig. S17) are marked by vertical lines.

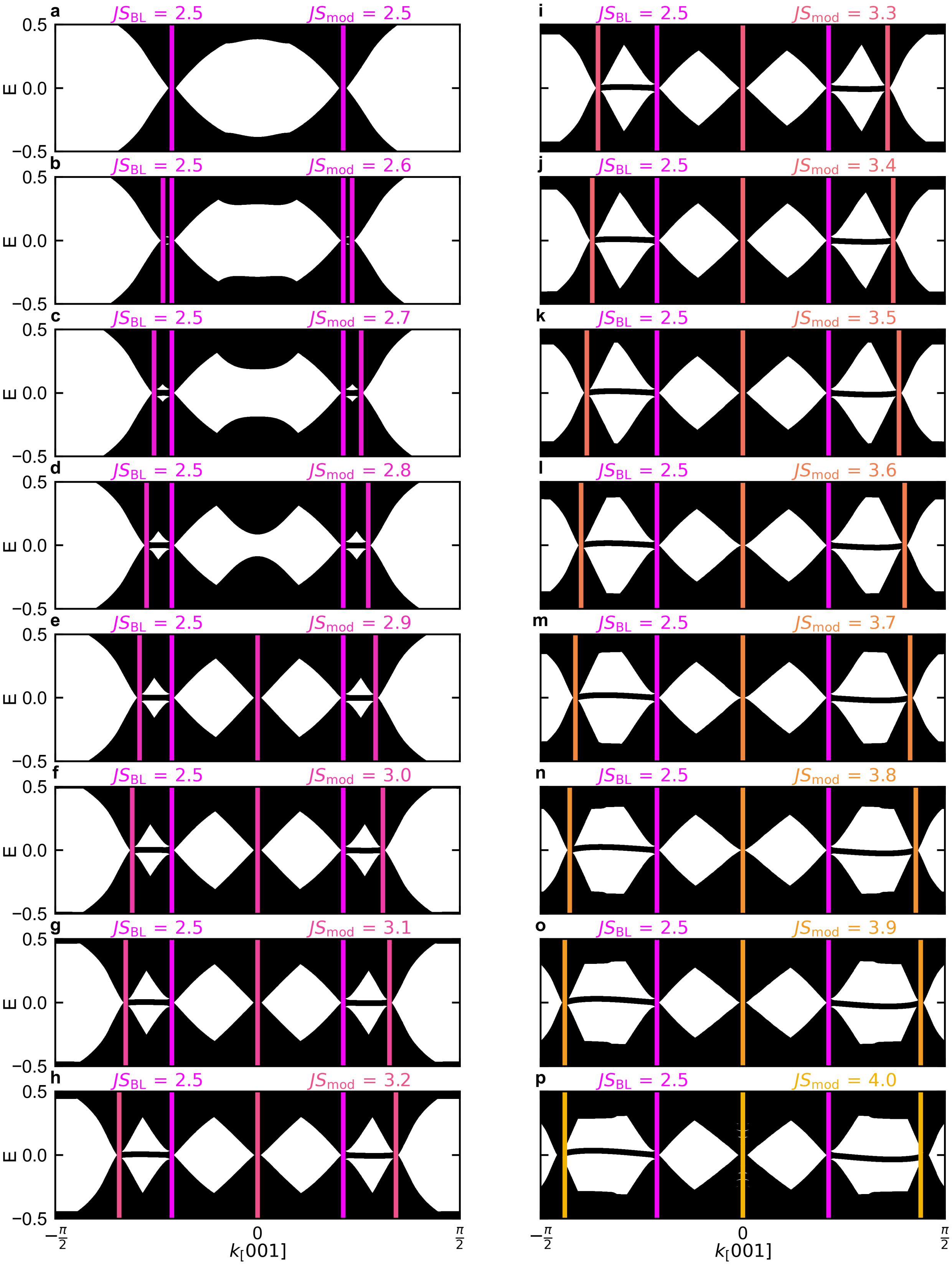

FIG. S19. **Band structures for a stripe geometry with an ↑↓ edge for alternating domains of BL and a modified layer with varied** $2.5 \leq JS_{\mathrm{mod}} \leq 4.0$**.** (**a-p**) The value of JS_{mod} is varied in 0.1 increments. The stripe is oriented along the [001] direction. The position of the BL bulk nodal points and of the bulk nodal points of the modified layer (cf. Fig. S17) are marked by vertical lines.

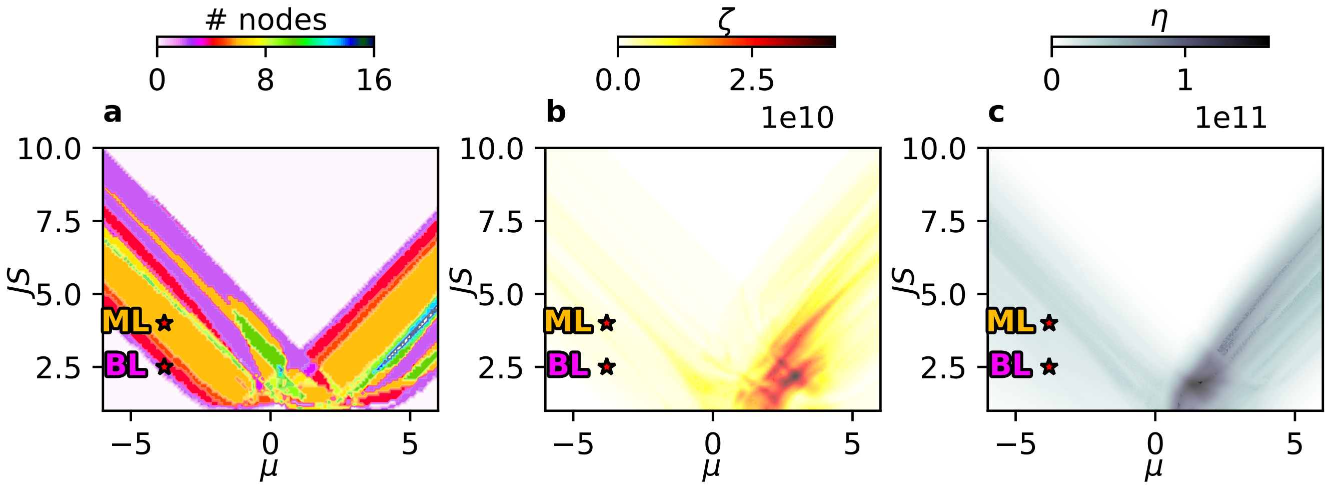

FIG. S20. **Nodal points and number of in-gap states in the tight-binding model for different parameters.** The chemical potential and the coupling to the magnetic state are varied between $-6 \leq \mu \leq 6$ and $1 \leq JS \leq 10$. **a** Number of nodal points in the BZ. **b** Difference ζ for a AFM state oriented along the $[110]$ and the $[001]$ direction. **c** Square of LDOS η within the Ta(110) bandgap for a AFM state in $[110]$ direction. The stars mark the parameters used for the ML and the BL.

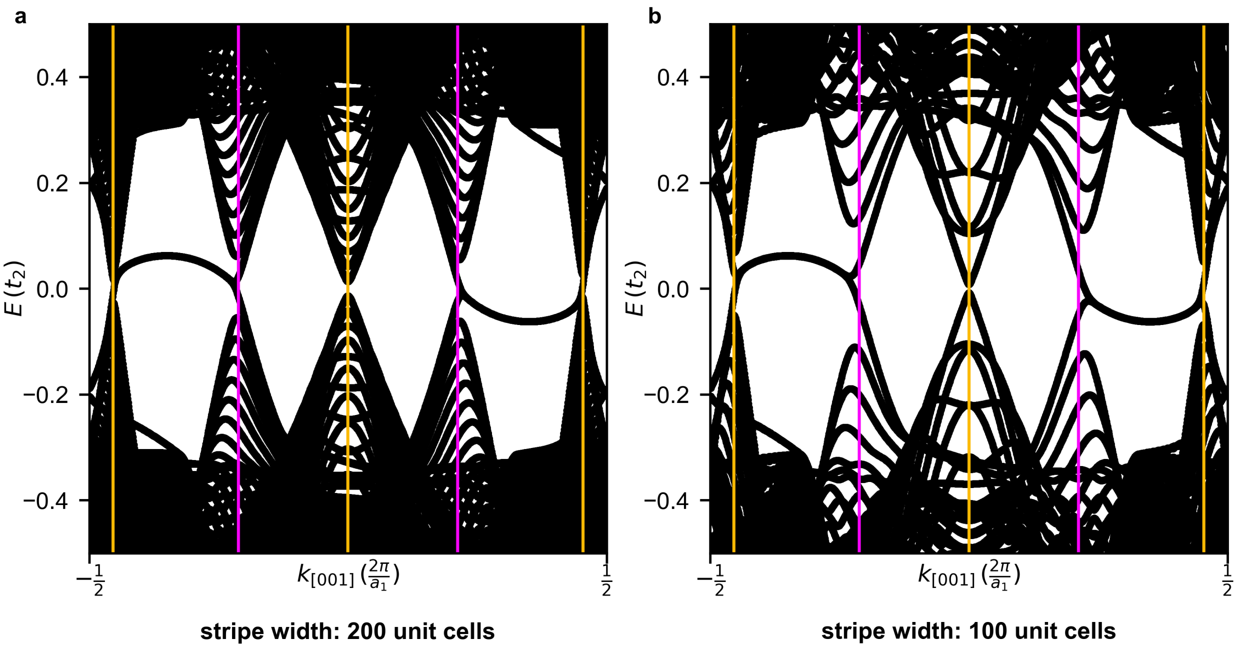

FIG. S21. **Convergence test for band structures in dependency of the stripe width for an ↑↑ edge.** The width of each BL or ML stripe is **a** 200 unit cells and **b** 100 unit cells. All other parameters of the calculations are the same. The position of the bulk nodal points are marked by vertical lines. The band structures of both stripes show nodal points at the position of the bulk nodal points, indicating bulk-like properties within the stripe. The edge modes for both stripe width are very similar, indicating no interaction between edge modes across the stripe, as these interactions would need to decay exponentially with the stripe width.

* Email: felix.zahner@uni-hamburg.de
† Email: nickel@physik.uni-kiel.de

[1] B. Santos, J. M. Puerta, J. I. Cerda, R. Stumpf, K. von Bergmann, R. Wiesendanger, M. Bode, K. F. McCarty, and J. de la Figuera, Structure and magnetism of ultra-thin chromium layers on W(110), New Journal of Physics **10**, 013005 (2008).

[2] R. Brüning, J. Bedow, R. Lo Conte, K. von Bergmann, D. K. Morr, and R. Wiesendanger, The Noncollinear Path to Two-Dimensional Topological Superconductivity, ACS Nano **19**, 36215 (2025).

[3] See `https://www.vasp.at`.

[4] G. Kresse and J. Furthmüller, Efficient iterative schemes for ab initio total-energy calculations using a plane-wave basis set, Phys. Rev. B **54**, 11169 (1996).

[5] G. Kresse and D. Joubert, From ultrasoft pseudopotentials to the projector augmented-wave method, Phys. Rev. B **59**, 1758 (1999).

[6] P. E. Blöchl, Projector augmented-wave method, Phys. Rev. B **50**, 17953 (1994).

[7] J. P. Perdew, K. Burke, and M. Ernzerhof, Generalized gradient approximation made simple, Phys. Rev. Lett. **77**, 3865 (1996).

[8] M. Bazarnik, R. Lo Conte, E. Mascot, K. von Bergmann, D. K. Morr, and R. Wiesendanger, Antiferromagnetism-driven two-dimensional topological nodal-point superconductivity, Nat. Commun. **14**, 614 (2023).

[9] J. Bedow, E. Mascot, T. Posske, G. S. Uhrig, R. Wiesendanger, S. Rachel, and D. K. Morr, Topological superconductivity induced by a triple-q magnetic structure, Physical Review B **102**, 180504(R) (2020).

[10] F. Nickel and S. Heinze, Topological properties of magnet-superconductor hybrid systems due to atomic-scale non-coplanar spin textures, npj Spintronics **3**, 13 (2025).
